# MEK inhibition is a promising therapeutic strategy for *MLL*-rearranged infant acute lymphoblastic leukemia patients carrying *RAS* mutations

**DOI:** 10.18632/oncotarget.11730

**Published:** 2016-08-31

**Authors:** Mark Kerstjens, Emma M.C. Driessen, Merel Willekes, Sandra S. Pinhanços, Pauline Schneider, Rob Pieters, Ronald W. Stam

**Affiliations:** ^1^ Department of Pediatric Oncology/Hematology, Erasmus MC-Sophia Children's Hospital, Rotterdam, The Netherlands; ^2^ Princess Máxima Center for Pediatric Oncology, Utrecht, The Netherlands

**Keywords:** MLL-rearrangements, RAS-pathway, leukemia, MEK inhibitors

## Abstract

Acute lymphoblastic leukemia (ALL) in infants is an aggressive malignancy with a poor clinical outcome, and is characterized by translocations of the *Mixed Lineage Leukemia (MLL)* gene. Previously, we identified *RAS* mutations in 14-24% of infant ALL patients, and showed that the presence of a *RAS* mutation decreased the survival chances even further. We hypothesized that targeting the RAS signaling pathway could be a therapeutic strategy for *RAS*-mutant infant ALL patients. Here we show that the MEK inhibitors Trametinib, Selumetinib and MEK162 severely impair primary *RAS*-mutant *MLL*-rearranged infant ALL cells *in vitro*. While all *RAS*-mutant samples were sensitive to MEK inhibitors, we found both sensitive and resistant samples among *RAS*-wildtype cases. We confirmed enhanced RAS pathway signaling in *RAS*-mutant samples, but found no apparent downstream over-activation in the wildtype samples. However, we did confirm that MEK inhibitors reduced p-ERK levels, and induced apoptosis in the *RAS*-mutant *MLL*-rearranged ALL cells. Finally, we show that MEK inhibition synergistically enhances prednisolone sensitivity, both in *RAS*-mutant and *RAS*-wildtype cells. In conclusion, MEK inhibition represents a promising therapeutic strategy for *MLL*-rearranged ALL patients harboring *RAS* mutations, while patients without *RAS* mutations may benefit through prednisolone sensitization.

## INTRODUCTION

Acute lymphoblastic leukemia (ALL) in infants (<1 year of age) represents an aggressive malignancy, associated with high relapse rates and a poor clinical outcome [1]. The majority (~80%) of these patients carry a leukemia-specific chromosomal translocations involving the *Mixed Lineage Leukemia* (*MLL*) gene [1]. *MLL*-rearranged infant ALL patients fare significantly worse than infant ALL patients who do not carry *MLL* translocations, with event-free survival rates of 30-40% vs. ~80%, respectively [2]. Recently, we demonstrated that 24% of the infant ALL patients carrying *MLL* translocation t(4;11), the most frequently observed translocation of *MLL* among these patients, also carry a *RAS* mutation. Mutations in *NRAS* were found in 11% and *KRAS* mutations in 13% of cases [3]. Moreover, we showed that the presence of a *RAS* mutation in *MLL*-rearranged patients represented an independent predictive factor for an even worse clinical outcome in this high-risk group. Nearly all *RAS*-mutant t(4;11)^+^ infant ALL patients relapsed within the first year from diagnosis, while still on treatment, and all died within 4 years from diagnosis [3].

Despite this strong association with an exceedingly poor prognosis, a recent study by Emerenciano *et al*. suggested that *RAS* mutations in *MLL*-rearranged infant ALL may not act as driver mutations and are not required for disease progression, but rather act only at disease onset [4]. Yet, our previous data clearly showed that *RAS*-mutant *MLL*-rearranged infant ALL patients are at extremely high risk of therapy failure and early death. Moreover, RAS pathway inhibition, including MEK inhibition, was previously shown to effectively inhibit *RAS*-mutant *MLL*-rearranged AML *in vitro* [5, 6]. Therefore, we decided to investigate the potential of *RAS* pathway inhibition and found that *RAS*-mutant *MLL*-rearranged ALL cells are remarkably sensitive to MEK inhibitors.

## RESULTS

### *RAS*-mutant *MLL*-rearranged ALL cells are sensitive to MEK inhibition

**Figure 1:**
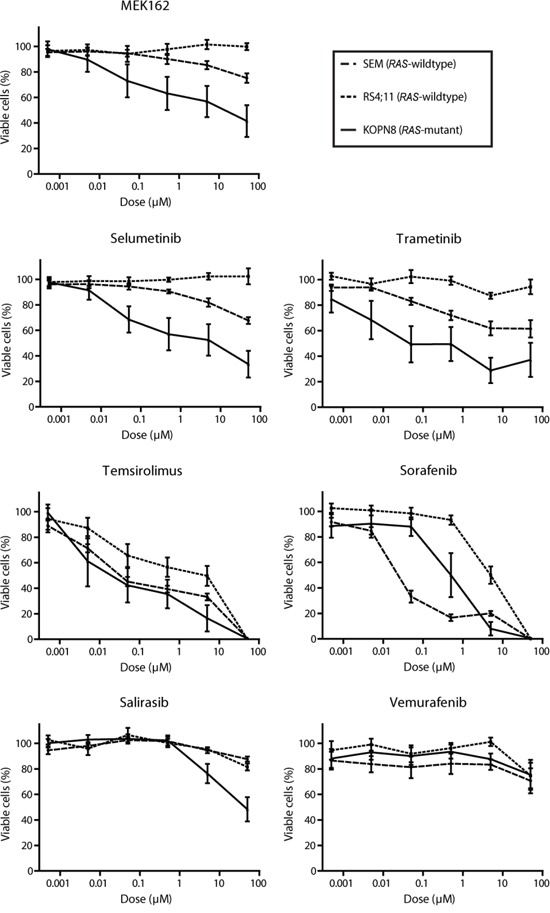
MEK inhibitors specifically impede *RAS*-mutant *MLL*-rearranged ALL cell line KOPN8 Cell viability of *MLL*-rearranged cell lines exposed to MEK162, Selumetinib, Trametinib, Temsirolimus, Sorafenib, Salirasib and Vemurafenib. All cell lines respond to Sorafenib and Temsirolimus, while *RAS*-mutant KOPN8 (solid line) is more sensitive for MEK162, Selumetinib and Trametinib than *RAS*-wildtype SEM (large dashed line) or RS4;11 (small dashed line). Data are represented as mean +/− sem. n≥3.

**Figure 2:**
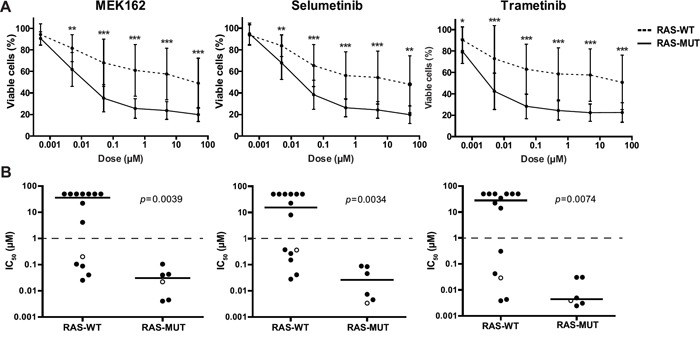
Primary *RAS*-mutant *MLL*-rearranged ALL cells are sensitive to MEK inhibitors **A**. Patient derived t(4;11)^+^ infant ALL cells exposed to MEK inhibitors indicate *RAS*-mutant samples (solid line, n=6) are more sensitive compared to *RAS*-wildtype samples (dashed line, n=14). Data are represented as median +/− sd. *0.01<*p*<0.05; **0.001<*p*<0.01; ***p<0.001. **B**. The IC_50_ (concentration needed to inhibit 50% of the leukemic cell viability) of the individual t(4;11)^+^ infant ALL patient samples shown in A. Median IC_50_ values, represented by horizontal bars, confirm strong sensitivity of *RAS*-mutant patient samples compared to the majority of *RAS*-wildtype samples. Open circles indicate matched diagnosis (wildtype) and relapse (mutant) samples. The tick lines indicate separation between MEK inhibitor sensitive and resistant patient samples (IC_50_<1μM and IC_50_>1μM, respectively).

Since the previously identified *RAS* aberrations are all activating mutations (at residues G12, G13 or Q61), we wondered whether small molecule inhibitors targeting RAS pathway components could suppress *RAS*-mutant leukemic cells [3, 7]. Therefore, 7 RAS pathway inhibitors, already approved for therapeutical use or under clinical investigation for other malignancies with RAS pathway mutations, were selected as therapeutic strategies for the *RAS*-mutant infant ALL patients. Using 4-day MTS cell viability assays we tested the *in vitro* anti-leukemic potential of Salirasib (RAS localization inhibitor), Vemurafenib (BRAF inhibitor), Sorafenib (pan-kinase inhibitor), Trametinib, Selumetinib and MEK162 (MEK inhibitors) and Temsirolimus (mTOR inhibitor) against *RAS*-mutant *MLL*-rearranged ALL cell line KOPN8, and the *RAS*-wildtype *MLL*-rearranged cell lines SEM and RS4;11. Interestingly, the *RAS*-mutant cell line KOPN8 was more sensitive to the MEK inhibitors MEK162, Selumetinib and Trametinib (Figure 1). Temsirolimus and Sorafenib potently reduced cell viability of both *RAS*-mutant and *RAS*-wildtype cell lines. Additionally, Salirasib and Vemurafenib did not substantially reduce cell viability, even at high concentrations (>10μM). To confirm the efficacy of these inhibitors, we performed 4-day MTT cell viability assays on primary diagnostic *RAS*-mutant (n=6) and *RAS*-wildtype (n=14) t(4;11)^+^ infant ALL samples. Interestingly, compared to *RAS*-wildtype t(4;11)^+^ ALL cases, the *RAS*-mutant t(4;11)^+^ infant ALL cases were significantly more sensitive to all MEK inhibitors (Figure 2A) with median IC_50_ values of <0.1 μM for MEK162 and Selumetinib and <0.01 μM for Trametinib (Figure 2B). Additionally, all other tested inhibitors (Salirasib, Temsirolimus, Sorafenib and Vemurafenib) reached only IC_50_ values of >10μM (Supplementary Figure 1).

Also, we included one matched pair of diagnostic/relapse t(4;11)^+^ samples. For this particular patient, no *RAS* mutation was present at diagnosis, but a *RAS* mutation could be identified at relapse. Indeed, the *RAS*-mutant relapse sample of this patient was more sensitive to all three MEK inhibitors tested than the *RAS*-wildtype diagnostic sample (Figure 2B).

### Enhanced RAS activation in t(4;11)^+^ infant ALL cells carrying *RAS* mutations

**Figure 3:**
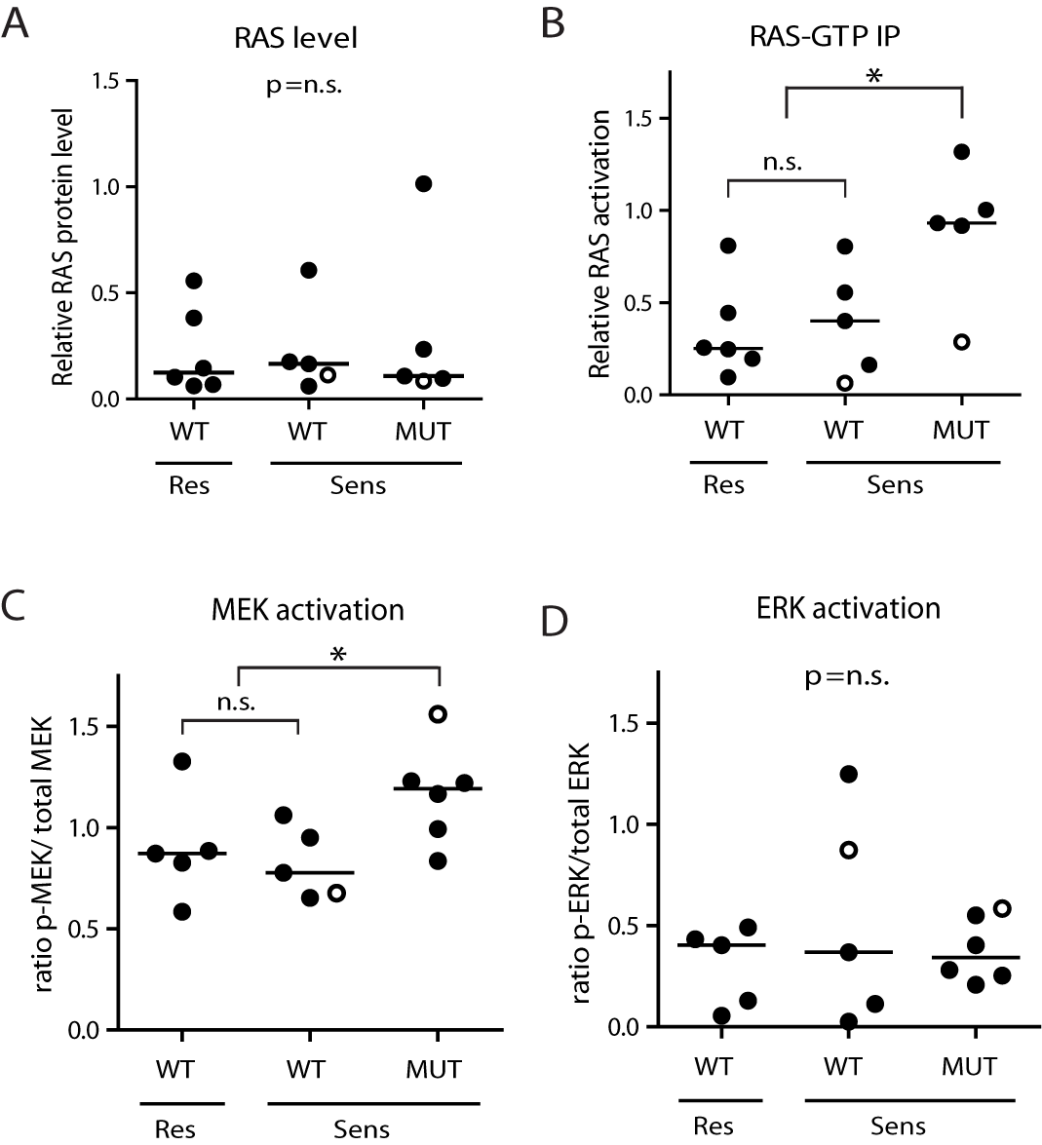
*RAS*-mutant t(4;11)-positive ALL cells have enhanced downstream activation **A**. RAS protein level, relative to β-actin, determined by western blotting in t(4;11)^+^ infant ALL samples, subdivided according to *RAS* mutation status (WT or MUT) and MEK inhibitor sensitivity (Res or Sens). No differences in median protein level (horizontal bars) are observed between the different subgroups. **B**. Relative RAS activation is enhanced in *RAS*-mutant t(4;11)^+^ patient samples, though no difference is observed between the MEK inhibitor resistant and sensitive *RAS*-wildtype subgroups. **C**. Ratio p-MEK/total MEK in *RAS*-mutant (MUT) and *RAS*-wildtype (WT) t(4;11)-rearranged infant ALL samples shows increased MEK activation in *RAS*-mutant samples, while the MEK inhibitor resistant and sensitive *RAS*-wildtype samples have comparable MEK activation. **D**. Ratio p-ERK/total ERK in *RAS*-mutant and *RAS*-wildtype t(4;11)-rearranged infant ALL samples shows no significant differences in ERK activation between subgroups. Open circles indicate matched diagnosis (wildtype) and relapse (mutant) samples. Horizontal bars present group medians. Open circles indicate matched diagnosis (wildtype) and relapse (mutant) samples. *p<0.05.

The MEK inhibitors MEK162, Selumetinib and Trametinib significantly reduce viability of *RAS*-mutant *MLL*-rearranged ALL cells. Notably, a subset of the *RAS*-wildtype primary t(4;11)^+^ infant ALL samples also responded favorably to the MEK inhibitors (Figure 2B). We wondered whether other biomarkers, besides *RAS* mutation status, could predict MEK inhibitor sensitivity in *MLL*-rearranged ALL. Wildtype RAS proteins are under regulation of upstream signaling events, often involving tyrosine kinase receptors, while mutant RAS proteins are less dependent on upstream activation due to reduced GTPase activity, rendering a surplus of activated GTP-bound RAS. Therefore, we determined the RAS protein levels and RAS activity in our primary t(4;11)^+^ infant ALL cells. No significant difference in RAS protein levels was observed between the *RAS*-mutant and *RAS*-wildtype t(4;11)^+^ infant ALL samples using Western blot analysis (Figure 3A). Next, we investigated the level of active (GTP-bound) RAS in these samples by precipitation with RAF-1 RAS interaction peptide, followed by immunoblotting. As expected, the *RAS*-mutant t(4;11)^+^ infant ALL samples showed significant (*p*=0.013) higher levels of RAS activation as compared to *RAS*-wildtype samples (Figure 3B). No differences in RAS activation were observed between *RAS*-wildtype samples that were sensitive or resistant to MEK inhibition.

Subsequently, we determined phosphorylation levels of MEK (p-MEK) and ERK (p-ERK) by immunoblotting (Supplementary Figure 2A and 2B, respectively). Quantification of the blots indicated a significantly higher level of p-MEK in our *RAS*-mutant samples, compared to *RAS*-wildtype samples (*p*=0.0312, Figure 3C), although there was no difference in p-MEK levels between the MEK inhibitor resistant and sensitive *RAS*-wildtype subgroups. Still, we did find a higher p-MEK level in the mutated relapse sample compared to its matched wildtype diagnosis sample. Additionally, no differences in p-ERK levels were found between *RAS*-wildtype and *RAS*-mutant samples (Figure 3D), nor between *RAS*-wildtype cells that were sensitive or resistant to MEK inhibition.

In *MLL*-rearranged AML, MEK inhibitor resistance can occur through activation of tyrosine kinase receptor (TKR) signaling (i.e. involving VEGFR-2) [5]. Furthermore, we previously found *MLL*-rearranged ALL is characterized by high expression of Fms-like tyrosine kinase 3 (*FLT3*) [8]. Therefore, we interrogated available gene expression profiles of primary samples for possible differences in TKR expression levels between the MEK inhibitor sensitive and resistant subgroups (Supplementary Figure 3). Interestingly, apart from *FLT3*, expression of TKRs is relatively low in the different patient samples. Surprisingly, *FGFR-1* expression is significantly lower in MEK inhibitor resistant *RAS*-wildtype samples (*p*=0.02), while there are no significant differences in expression of *FLT3*, *VEGFR* (1-3), *FGFR* (2-4), *EGFR* and *ERBB* (2-4), *PDGFR* (A-B) or *Lck* and *Src*.

### MEK inhibition results in reduced ERK phosphorylation

**Figure 4:**
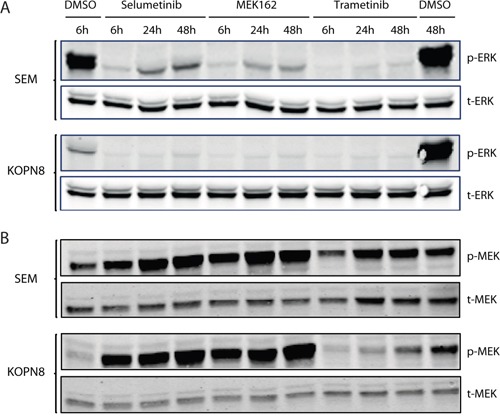
MEK inhibition results in reduced ERK phosphorylation **A**. Western blot analysis of SEM and KOPN8 (upper and lower panels, respectively) exposed to 500 nM of MEK inhibitor or vehicle control (DMSO) for 6, 24 and 48 hours. Both cell lines almost completely lose ERK phosphorylation (p-ERK), while total ERK (t-ERK) levels remain unaffected. **B**. Analysis of MEK phosphorylation (p-MEK) suggests exposure to MEK162 and Selumetinib results in enhanced MEK phosphorylation in both cell lines, whereas total MEK (t-MEK) levels remain constant.

Next, we exposed *MLL*-rearranged ALL cell lines SEM and KOPN8 to the MEK inhibitors (Selumetinib, MEK162 and Trametinib) and determined p-ERK and p-MEK levels by immunoblotting (Figure 4). Interestingly, p-ERK levels were drastically reduced in both SEM and KOPN8, already after 6 hours of exposure, and this effect was sustained for at least 48 hours, regardless of the inhibitor used (Figure 4A). Furthermore, prolonged exposure (24 and 48 hours) to the MEK inhibitors Selumetinib and MEK162 resulted in an increase of p-MEK in SEM and KOPN8 (Figure 4B). Additionally, we determined phosphorylation of ERKs downstream effector ELK-1, but ELK-1 activation was not influenced by MEK inhibition (Supplementary Figure 4A).

Since SEM cells responded modestly to MEK inhibition but did show a significant loss of p-ERK levels, we investigated whether these cells could circumvent loss of ERK activation by upregulating RAS-mediated PI3K-Akt-mTOR signaling. Therefore, the downstream phosphorylation of Akt (Ser437) and p70S6K (Thr389) was assessed by immunoblotting. However, no differences in Akt and p70S6K phosphorylation were observed in response to MEK inhibitor exposure (Supplementary Figure 4B and 4C).

### MEK inhibitors induce apoptosis

**Figure 5:**
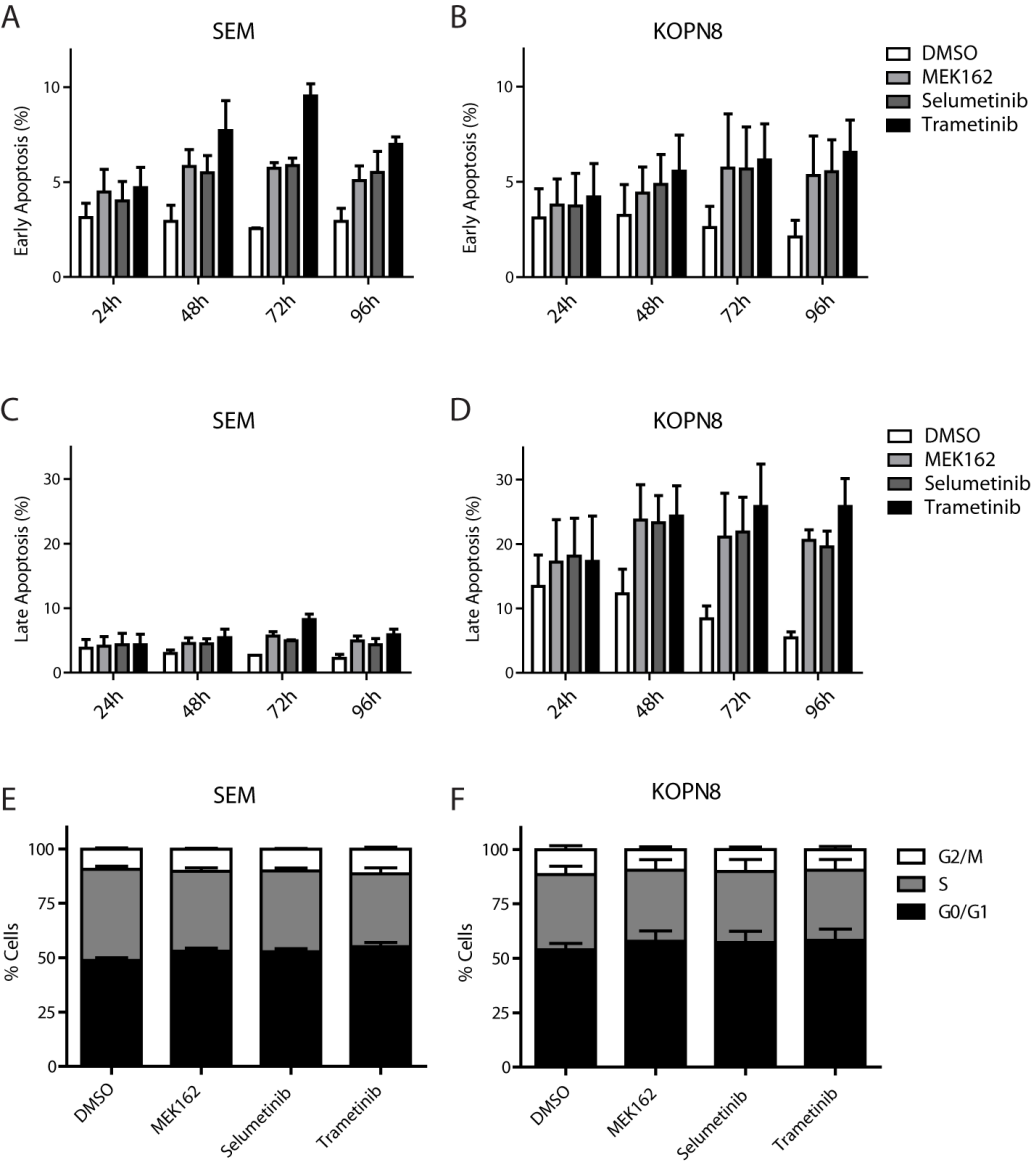
MEK inhibitors induce apoptosis **A**. and **B**. Early apoptosis (percentage AnnexinV single positive of total) of SEM and KOPN8 cells (respectively) after exposure to DMSO vehicle (white bars) or 500 nM MEK162, Selumetinib or Trametinib (light grey, dark grey and black bars, respectively), indicates MEK inhibition slightly induces early apoptosis. Data are represented as mean +/− sem. n=3. **C**. and **D**. Late apoptosis (percentage AnnexinV and 7-AAD double positive cells of total) of SEM and KOPN8 (respectively) show that while SEM cells have no induction of late apoptosis in response to MEK inhibition, compared to the DMSO controls, KOPN8 clearly undergoes apoptosis, especially after prolonged exposure (>48 hours). Data are represented as mean +/− sem. n=3. **E**. and **F**. Cell cycle analysis of SEM and KOPN8 (respectively) after 96 hours exposure to vehicle (DMSO) or 500 nM MEK162, Selumetinib or Trametinib indicates MEK inhibition does not impinge on the cell cycle progression. Stacked bar graph indicates percentage of cells in G_0_/G_1_ (black), S (grey) and G_2_/M (white) cell cycle stages. Data are represented as mean +/− sem. n=3.

Subsequently, we investigated the phenotypic effects of the MEK inhibitors on SEM and KOPN8 through analysis of early and late apoptosis markers (Annexin-V and 7-AAD, respectively), using flow-cytometry. Interestingly, both *RAS*-wildtype SEM and *RAS*-mutant KOPN8 undergo early apoptosis, after treatment with MEK inhibitor (Figure 5A and 5B, respectively). However, while late apoptosis is barely observed for SEM (Figure 5C), late apoptosis in MEK inhibitor exposed KOPN8 cells is enhanced substantially, especially after prolonged exposure (Figure 5D), suggesting the response to MEK inhibition is characterized by increased apoptosis. Furthermore, MEK inhibitor exposure induced protein levels of pro-apoptotic BIM, most evidently for KOPN8, while p53 levels remained unaffected (Supplementary Figure 5). Additionally, we investigated cell cycle progression under influence of MEK inhibition. Interestingly, no considerable differences in SEM or KOPN8 cell cycle progression were observed after 96 hours exposure to MEK162, Selumetinib or Trametinib (Figure 5E and 5F, respectively), nor after exposure for 24, 48 and 72 hours (Supplementary Figure 6A-6B, 6C-6D and 6E-6F, respectively).

### MEK inhibition enhances prednisolone sensitivity

**Figure 6:**
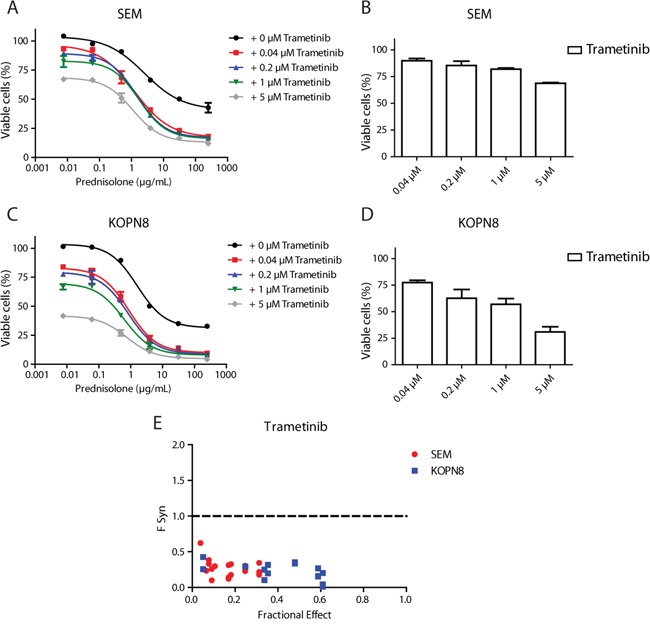
MEK inhibition enhances prednisolone sensitivity **A**. Dose-response curves of the SEM cell line exposed to prednisolone alone (black curve) or in combination with 0.04 μM, 0.2 μM, 1 μM or 5 μM Trametinib (red, blue, green and grey curves, respectively). Low concentrations of Trametinib particularly sensitize cells that escape high concentrations of prednisolone. Data are represented as mean +/− sem. n=3. **B**. Response of SEM to the single Trametinib concentrations used in A. n=3. **C**. Dose-response curves of KOPN8 treated with prednisolone (black curve), or in combination with the aforementioned Trametinib concentrations (shown in red, blue, green and grey, respectively). KOPN8 cells are also sensitized towards prednisolone by co-exposure with low concentrations of Trametinib. Data are represented as mean +/− sem. n=3 **D**. KOPN8 exposed to single Trametinib concentrations. Data are represented as mean +/− sem. n=3. **E**. Combined exposure to prednisolone and Trametinib (merged data from 3 separate experiments) was quantified using F_Syn_ calculations (F_Syn_<1 indicates synergy) and plotted against fractional effect (i.e. inhibition of cell viability). In SEM (red) moderate to strong synergy was observed, while all combinations of Trametinib and prednisolone result in strong to very strong synergy in KOPN8 (blue).

In our previous study, we found that *MLL*-rearranged infant ALL patient samples harboring *RAS* mutations are more resistant to prednisolone [3]. Therefore, we examined whether inhibition of MEK could enhance prednisolone sensitivity of *RAS*-mutant cells. As shown in Figure 6A, prednisolone alone decreased cell viability of SEM cells to only ~50%. Interestingly, while Trametinib by itself induced only minor cell viability decrease in SEM cells (Figure 6B), the combination of Trametinib and prednisolone greatly enhanced the efficacy of prednisolone, especially at higher concentrations (Figure 6A). The combination of prednisolone and Trametinib also strongly decreased cell viability in KOPN8 more potently than either drug alone; low concentrations of Trametinib nearly eradicated all KOPN8 cells that did not respond to prednisolone treatment (Figure 6C). A similar sensitizing effect was observed when exposing SEM and KOPN8 to MEK162 or Selumetinib in combination with prednisolone (Supplementary Figure 7A-7D and 7F-7I, respectively). Since Trametinib alone already effectively decreases viability of KOPN8 cells (Figure 6D), we quantified the combinatorial effect of MEK inhibitors and prednisolone using the synergy factor (F_Syn_) calculation, as previously described [9, 10]. The plot in Figure 6E shows the fractional effect (i.e. the relative decrease of cell viability) induced by the combination of Trametinib with prednisolone, and the corresponding Synergy Factor. Interestingly, in both SEM and KOPN8 cells we observed F_Syn_ values < 0.5, indicating strong synergy between Trametinib and prednisolone. Also combining MEK162 or Selumetinib with prednisolone resulted in moderate to strong synergistic effects (Supplementary Figure 7E and 7J, respectively). Additionally, we investigated whether this enhanced effect was related to differential expression of the glucocorticoid receptor (GR), the target of prednisolone. However, MEK inhibitor exposure did not alter GR protein levels in either SEM or KOPN8 cells (Supplementary Figure 7K).

## DISCUSSION

*MLL*-rearranged ALL in infants is a high-risk hematologic malignancy, characterized by a high incidence of relapse and high mortality rate [11]. Recently, we showed that 14-24% of these patients carry a *RAS* mutation, as an independent predictor of extremely poor outcome [3]. In the present study, we demonstrate that the MEK inhibitors Trametinib, Selumetinib and MEK162 display strong anti-leukemic effects against *RAS*-mutant *MLL*-rearranged ALL cells. Considering the dismal prognosis for infants suffering from *MLL*-rearranged ALL with additional *RAS* mutations, our data supports application of these inhibitors in the treatment of this patient group. Recently, Irving *et al*. already showed that Selumetinib effectively inhibits leukemia progression in an *in vivo* model of *RAS*-mutant BCP-ALL, and Burgess *et al*. found Trametinib to prolong the survival of mice transplanted with *NRAS*^G12D^ AML cells [12, 13]. Moreover, Trametinib has recently been approved for the treatment of adult *BRAF*-mutated melanoma, while different clinical trials with Selumetinib and MEK162 show promising results in adult patients with *RAS*/*RAF* mutation positive melanoma and non-small-cell lung cancer [14–18]. Even though most clinical trials focused on solid tumors in adult patients, pediatric clinical trials are underway for neurofibromas and gliomas, and could expedite clinical application of these MEK inhibitors in *MLL*-rearranged infant ALL.

Interestingly, while all *RAS*-mutant *MLL*-rearranged ALL patient samples are susceptible to MEK inhibition, patients without *RAS* mutations also might benefit from MEK inhibitor treatment, since a subgroup of *RAS*-wildtype patient samples appears sensitive to MEK inhibition. While in our previous study, we identified *RAS* mutations and found no *BRAF* aberrations, mutations of other upstream regulators, i.e. tyrosine kinase receptors, can occur in other malignancies [3]. Andersson *et al*. recently showed that additional somatic mutations in *MLL*-rearranged infant ALL, like (sub-)clonal RAS/PI3K pathway aberrations, occur in up to 50% of the cases, supporting our previous observation that *RAS* mutations in *MLL*-rearranged infant ALL frequently occur at a sub-clonal level [3, 19]. These findings do not support the hypothesis that other (upstream) mutations are driving RAS-MEK-ERK signaling, but also do not explain observed extensive MEK inhibitor sensitivity of all (subclonal) *RAS*-mutant and specified *RAS*-wildtype patient samples. While we found enhanced RAS and MEK activation in *RAS*-mutant samples, these biomarkers could not differentiate MEK inhibitor sensitive and resistant *RAS*-wildtype samples. Interestingly, Kampen *et al*. recently proposed a MEK inhibitor escape mechanism in *MLL*-rearranged AML, which was mediated by VEGFR-2 and PI3K-signaling, and we wondered whether this could play a role in the MEK inhibitor resistance of our wildtype patient cells [5]. However, we observed no difference in downstream PI3K-signalling (i.e. Akt or p70S6K phosphorylation) in response to MEK inhibitor exposure. Additionally, we discovered no significant tyrosine kinase receptor expression differences in *MLL*-rearranged infant ALL patient samples that could explain the MEK inhibitor response of *RAS*-wildtype samples. Surprisingly, *FGFR-1* expression was lower in MEK inhibitor resistant samples, but it is unclear how this would explain MEK inhibitor resistance. Alternatively, Minjgee *et al*. report that *RAS*-mutant transfected cells can induce downstream RAS signaling in a paracrine manner, through excretion of cytokines [20]. Interestingly, Nakanishi *et al*. previously demonstrated that *MLL*-fusion proteins can induce ERK phosphorylation through regulating EphA7 receptor tyrosine kinase expression, but this was not accompanied by increased RAF or MEK phosphorylation [21]. Still, their data shows that leukemic cells carrying the t(4;11) translocation are sensitive to small molecule inhibitors of ERK phosphorylation. These findings indicate alternative regulatory mechanisms for ERK signaling in *MLL*-rearranged leukemia could explain the MEK inhibitor sensitivity we observe in *RAS*-wildtype cells.

Loss of ERK phosphorylation in response to MEK162, Selumetinib or Trametinib exposure confirmed the effect of MEK inhibition. Interestingly, prolonged exposure of cells to MEK162 or Selumetinib resulted in increased MEK phosphorylation. Previously, Hatzivassiliou *et al*. showed that the aromatic fluorine of allosteric MEK inhibitor GDC-0973 interacts with MEK residue S212 [22]. Their data indicate this interaction results in exposure of the phosphorylation sites S218/S222, which are then susceptible to RAF mediated phosphorylation. Since MEK162 and Selumetinib both have this aromatic fluorine, the mechanism of interaction with MEK is probably similar to GDC-0973. Hence, although MEK activation in presence of GDC-0973, MEK162 or Selumetinib can still occur, the transduction of the signal by MEK-mediated phosphorylation of ERK is no longer possible, as we show in Figure 4.

Recently, we found the presence of *RAS* mutations in *MLL*-rearranged infant ALL cells correlated with prednisolone resistance, an obstacle in the treatment of infant ALL [2, 3]. Remarkably, our present data shows that MEK inhibition strongly enhances the sensitivity of both *RAS*-wildtype and *RAS*-mutant *MLL*-rearranged ALL cells to prednisolone, also further exemplifying the possible value of MEK inhibitors for *RAS*-mutant, as well as *RAS*-wildtype, *MLL*-rearranged infant ALL patients. The prednisolone-sensitizing effect of MEK inhibitors proposes a possible role for RAS-MEK-ERK signaling in the response to glucocorticoids. Recent work by Jones *et al*. shows that MEK plays a key role in drug resistance in relapsed pediatric ALL, and that MEK inhibition can sensitize ALL relapse samples to chemotherapeutics, including methylprednisolone [23]. Moreover, Ariës *et al*. found Trametinib could restore prednisolone sensitivity in *RAS*-mutant BCP-ALL patient samples, whereas Rambal *et al*. showed that MEK activation reduces dexamethasone sensitivity, and the MEK inhibitor PD183452 enhanced dexamethasone responses in ALL cells in a BIM-dependent manner [24, 25]. Activated ERK can phosphorylate BIM, targeting it for proteasomal degradation, and thereby diminishing apoptosis induced by dexamethasone [26]. Moreover, we established that, while glucocorticoid receptor expression remains constant, MEK inhibition upregulates pro-apoptotic BIM, which implies that inhibiting MEK, resulting in abrogation of ERK phosphorylation, may result in prolonged maintenance of pro-apoptotic BIM activity upon prednisolone exposure, leading to enhanced prednisolone sensitivity. This is further supported by our previous study showing that in *MLL*-rearranged ALL, prednisolone sensitization mediated by pan-BCL-2 family inhibitors was largely driven by the up-regulation of pro-apoptotic BID and BIM [27].

In summary, our data shows that *RAS*-mutant *MLL*-rearranged infant ALL patients may benefit from therapeutic strategies administering small-molecule MEK inhibitors. Furthermore, since MEK inhibition sensitizes *MLL*-rearranged ALL cells to prednisolone regardless of the *RAS* mutations status, *RAS*-wildtype *MLL*-rearranged infant ALL patients may also benefit from MEK inhibitor treatment through enhanced sensitivity to prednisolone.

## MATERIALS AND METHODS

### Patient samples and cell lines

Bone marrow and peripheral blood samples from untreated infant ALL patients were collected at the Sophia Children's Hospital (Rotterdam, The Netherlands) as part of the international collaborative INTERFANT treatment protocol [2]. Approval for these studies was obtained from the Erasmus MC Institutional Review Board. Informed consent was obtained according to the Declaration of Helsinki. All samples were processed within 24 hours after sampling as described before, with optional removal of contaminating non-leukemic cells by immunomagnetic beads, to ensure leukemic blast content for all samples was >90% [8]. The t(4;11)-rearranged ALL cell line SEM and t(11;19)-rearranged ALL cell line KOPN8 were purchased from the German Collection of Microorganisms and Cell Cultures (DSMZ, Braunschweig, Germany), while the t(4;11)-rearranged ALL cell line RS4;11 was purchased from The Global Biosource Center (ATCC, Middlesex, UK). All cell lines were cultured in suspension in RPMI-1640 with GlutaMAX (Invitrogen Life Technologies, Waltham, MA, USA) supplemented with 10% Fetal Calf Serum, 100 IU/mL penicillin, 100 IU/mL streptomycin and 0.125 μg/mL amphotericin B (Invitrogen Life Technologies) at 37°C under 5% CO_2_ atmosphere.

### *In vitro* cytotoxicity assay and small molecule inhibitors

The *in vitro* cytotoxicity of MEK162, Selumetinib and Trametinib (MedChem Express, Stockholm, Sweden) was tested by MTS and MTT assays. All inhibitors were weighed, dissolved in dimethyl sulfoxide (DMSO) and stored at -20°C until use. Cytotoxicity assay dilutions were prepared in cell culture medium, keeping final DMSO concentration <0.5%. Final concentrations of the small molecule inhibitors ranged from 50 μM to 0.15 nM, indicated in the respective figures. The *in vitro* sensitivity of cell lines was assessed by using 4-day MTS conversion assays, as described previously [28]. *In vitro* cytotoxicity of patient cells was assessed by using a 4-day MTT conversion assay, as described before [8]. Data were normalized to vehicle (DMSO) controls.

### Western blot analysis

Protein extracts (25 μg) were electrophoretically resolved on pre-cast SDS-polyacrylamide gels (anyKD, TGX, Bio-Rad, Veenendaal, The Netherlands) and transferred to nitrocellulose membranes. Membranes were blocked with 5% bovine serum albumin and subsequently probed with antibodies directed against total or phosphorylated ERK, MEK, ELK-1, Akt, or p70S6K (Cell Signaling, Danvers, MA, USA). Membranes were counterstained with IRDye® 680/800 conjugated secondary antibodies (Li/COR, Leusden, The Netherlands) and were scanned by an Odyssey imaging system (Li/COR). Membranes were re-probed with mouse monoclonal anti-β-actin antibodies (Sigma-Aldrich, St. Louis, MO, USA) as loading control. Fluorescence was quantified using the Odyssey 3.0 application software.

### RAS activation

RAS activation was analyzed using the RAS Activation Assay Kit (17-218, Merck-Millipore, Amsterdam, The Netherlands). Briefly, 1×10^7^ cells were isolated and lysed with Mg^2+^ Lysis Buffer (MLB), and stored at -80°C until use. GST-fused RAF-1 RBD bead slurry was added to the lysate and incubated for 1 hour at 4°C while agitating. Beads were isolated by centrifugation and washed with MLB, and precipitated protein was denatured with Laemmli buffer at 95°C before immunoblotting. As a positive control, total cell lysate was included in the immunoblotting procedure. The provided RAS antibody (05-516, Merck-Millipore) was used, and GST (Cell Signaling) and β-actin (Sigma-Aldrich) antibodies were used as loading controls for the beads and total protein, respectively. Fluorescence was quantified using the Odyssey 3.0 application software.

### Annexin-V/7-AAD apoptosis and cell cycle assays

For assessment of early and late apoptosis, the PE Annexin-V Apoptosis Detection Kit (BD Pharmingen, Breda, The Netherlands) was used according to the manufacturer's protocol. Briefly, drug-exposed cells were isolated, washed with PBS and re-suspended in binding buffer. Cells were stained with PE Annexin V and/or 7-AAD for 15 minutes, and sorted using fluorescence activated cell sorting (FACS). Cell cycle progression was assessed by permeabilization of isolated cells through hypotonic lysis. Subsequently, RNAse treatment was performed, and DNA was stained using Propidium Iodide, after which FACS determined DNA content. Data was analyzed using FlowJo software (FlowJo, Ashland, OR, USA).

### Gene expression data

Recently published gene expression data (Affymetrix HU133plus2.0) for part of the t(4;11)^+^ patient samples was available (i.e. for 6 of 9 MEK inhibitor resistant *RAS*-wildtype samples, 4 of 5 sensitive *RAS*-wildtype samples and 3 of 6 *RAS*-mutant samples) [29]. This data is available in GEO database19 (accession number GSE19475) and was acquired as previously described [29]. Tyrosine kinase receptor expression was derived from this dataset, using the following probe sets: 206674_at (FLT3), 204406_at (VEGFR-1), 203934_at (VEGFR-2), 234379_at (VEGFR-3), 210973_s_at (FGFR-1), 208225_at (FGFR-2), 204380_s_at (FGFR-3), 204579_at (FGFR-4), 211551_at (EGFR), 210930_s_at (ERBB2), 226213_at (ERBB3), 214053_at (ERBB4), 205463_s_at (PDGFR-A), 217112_at (PDGFR-B), 204891_s_at (Lck) and 213324_at (Src).

### Statistical analysis

Statistical analyses were performed with SPSS Statistics version 17.0 (SPSS Inc. Chicago, IL, USA). All tests were two-tailed and *p*-values < 0.05 were considered significant. The effect of combining drugs (i.e. synergy, additivity or antagonism) was assessed using Berenbaums criteria, as previously described [9, 10]. Briefly, we calculated the Synergy Factor (F_Syn_) with the formula F_Syn_ = ([Drug X_in combination with Y_]/[Drug X]) + ([Drug Y_in combination with X_]/[Drug Y]) for a particular fractional effect. If the drug combination results in F_Syn_ < 1, this is considered synergy.

Members of the INTERFANT-99 study are as follows: M. Campbell (Programa Infantil Nacional de Drogas Atineoplasicas (PINDA)), M. Felice (Argentina), A. Ferster (Children's Leukemia Group (CLCG)), I. Hann and A. Vora (UK Children's Cancer Study Group (UKCCSG)), L. Hovi (Nordic Society of Paediatric Haematology and Oncology (NOPHO)), G. Janka-Schaub (Cooperative Study Group for Treatment of ALL (COALL)), C.K. Li (Hong Kong), G. Mann (Berlin-Frankfurt-Munster Group-Austria (BFM-A)), T. LeBlanc (French ALL Group (FRALLE)), R. Pieters (Dutch Childhood Oncology Group (DCOG)), G. de Rossi and A. Biondi (Associazione Italiana Ematologia Oncologia Pediatrica (AIEOP)), J. Rubnitz (St Jude Children's Research Hospital (SJCRH)), M. Schrappe (Berlin-Frankfurt-Munster Group-Germany (BFM-G)), L. Silverman (Dana-Farber Cancer Institute (DFCI)), J. Stary (Czech Paediatric Haematology (CPH)), R. Suppiah (Australian and New Zealand Children's Haematology/Oncology Group (ANZCHOG)), T. Szczepanski (Polish Paediatric Leukemia and Lymphoma Study Group (PPLLSG)), and M. Valsecchi and P. de Lorenzo (Trial Operating Center (CORS)).

We thank P. Garrido Castro and D. Geerts for scientific input and reading the manuscript.

## SUPPLEMENTARY MATERIALS FIGURES


