# Supplementary material for: MEK inhibition is a promising therapeutic strategy for MLL-rearranged infant acute lymphoblastic leukemia patients carrying RAS mutations
Source: Oncotarget. 2016 Aug 31;8(9):14835–46. doi: 10.18632/oncotarget.11730 (PMC5362448; doi:10.18632/oncotarget.11730)
Supplement: Supplementary file 1 [file oncotarget-08-14835-s001.pdf]

MEK inhibition is a promising therapeutic strategy for *MLL*-rearranged infant acute lymphoblastic leukemia patients carrying *RAS* mutations

SUPPLEMENTARY FIGURES

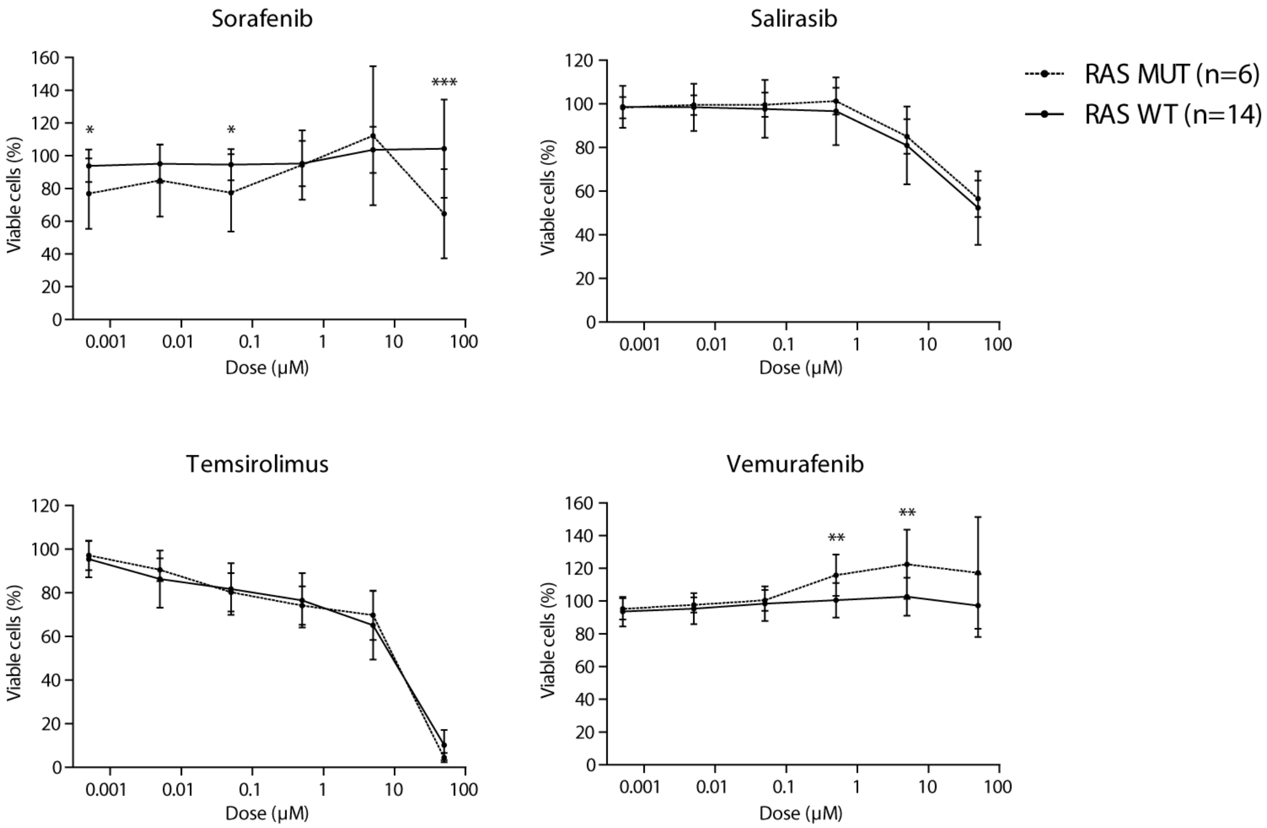

Supplementary Figure 1: MTT data primary t(4;11)$^{+}$ samples. MTT assays of patient derived *RAS*-mutant (solid line, n=6) and *RAS*-wildtype (dashed line, n=14) t(4;11)$^{+}$ infant ALL cells exposed to Sorafenib, Salirasib, Temsirolimus and Vemurafenib. Error bars represent standard deviation. *0.01<*p*<0.05; **0.001<*p*<0.01; ***p<0.001.

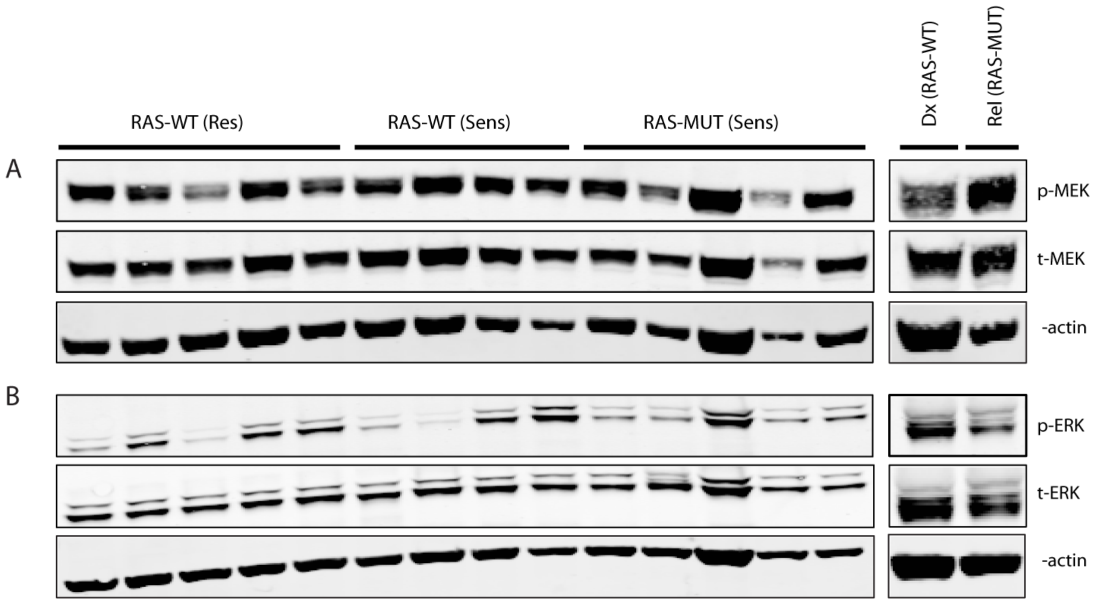

Supplementary Figure 2: p-MEK and p-ERK immunoblots of patient samples. A. Western blots of MEK inhibitor resistant (Res) and sensitive (Sens) *RAS*-wildtype and *RAS*-mutant t(4;11)$^+$ patient samples (left) and the matched diagnosis/relapse (Dx/Rel) samples (right) for phosphorylated MEK (upper), total MEK (middle) and β-actin (lower). **B.** Western blots of t(4;11)$^+$ patient samples for phosphorylated ERK (upper), total ERK (middle) and β-actin (lower).

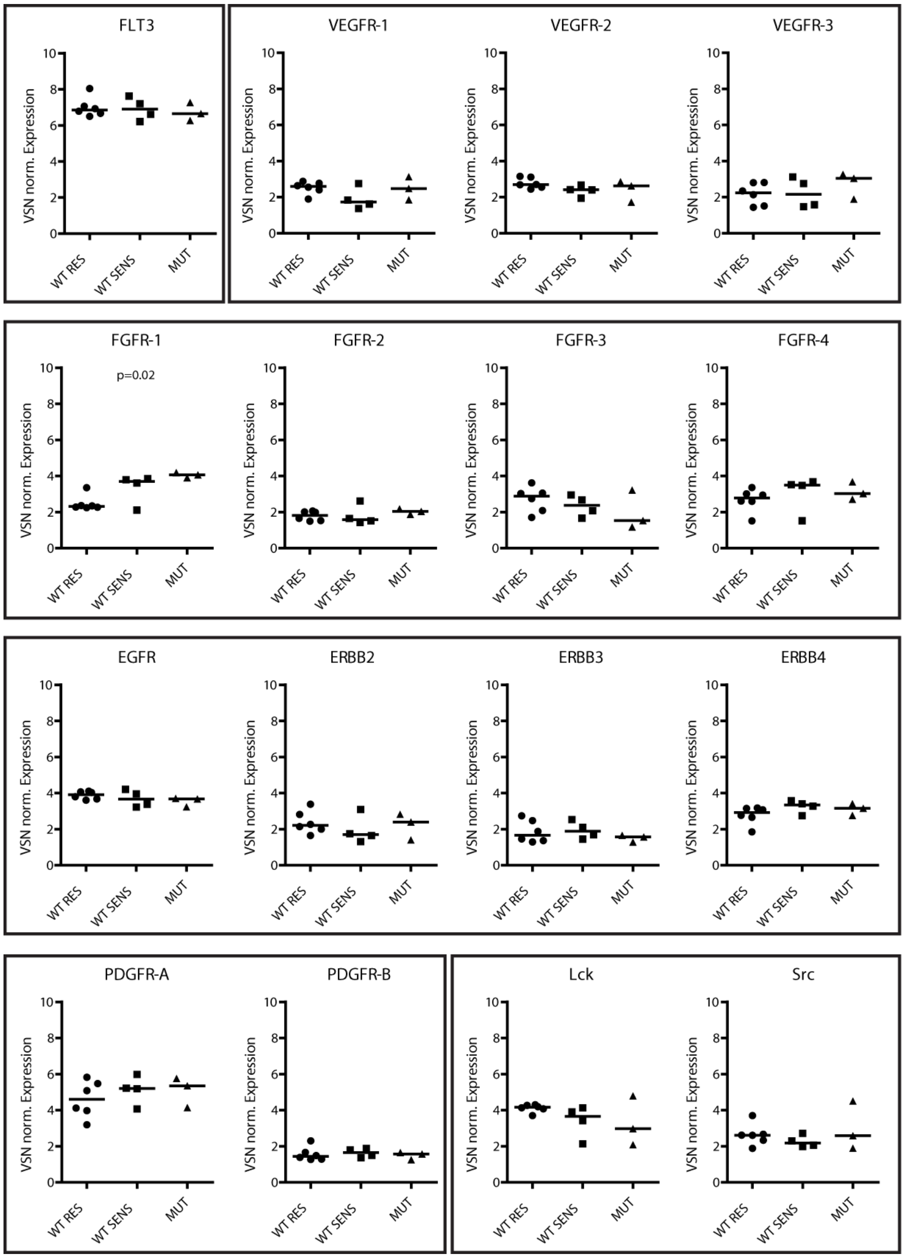

Supplementary Figure 3: Gene expression of tyrosine kinase receptors. Tyrosine kinase receptor mRNA expression (Affymetrix HU133plus2.0 microarray data) for FLT3, VEGFR-1, VEGFR-2, VEGFR-3, FGFR-1, FGFR-2, FGFR-3, FGFR-4, EGFR, ERBB2, ERBB3, ERBB4, PDGFR-A, PDGFR-B, Lck and Src in MEK inhibitor resistant *RAS*-wildtype (WT RES), MEK inhibitor sensitive *RAS*-wildtype (WT SENS) and *RAS*-mutant (MUT) primary samples.

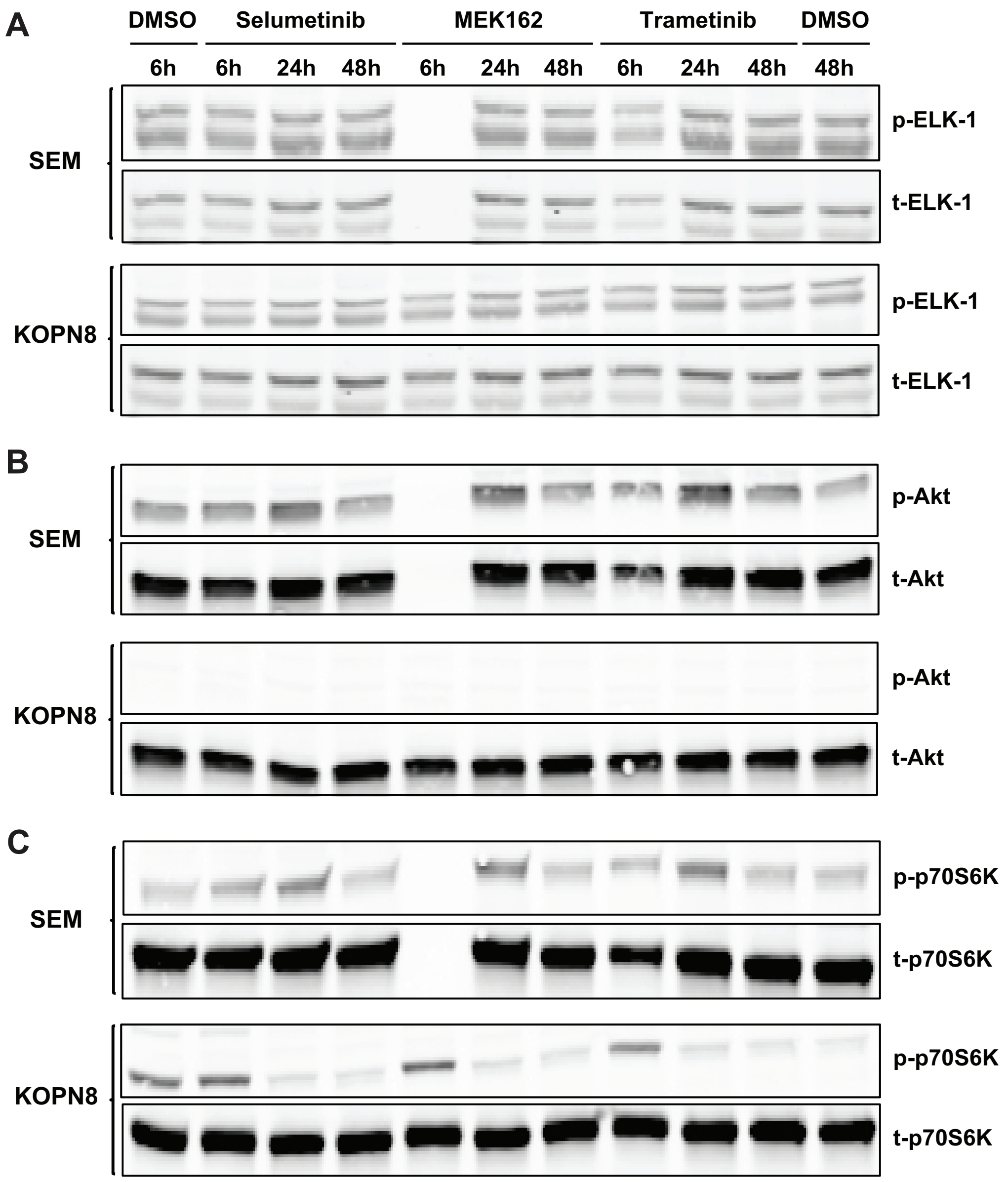

Supplementary Figure 4: p-ELK-1, p-Akt and p-p70S6K immunoblots of MEKi treated cells. A. Western blots of SEM (two upper panels) and KOPN8 (two lower panels), exposed for 6, 24 and 48 hours to either vehicle (DMSO) or Selumetinib, MEK162 or Trametinib, detecting phosphorylated ELK-1 and total ELK-1. **B.** Western blots of SEM and KOPN8, exposed to aforementioned MEK inhibitor conditions, for phosphorylated and total Akt. **C.** Western blots of SEM and KOPN8, exposed to aforementioned MEK inhibitor conditions, for phosphorylated and total p70S6K.

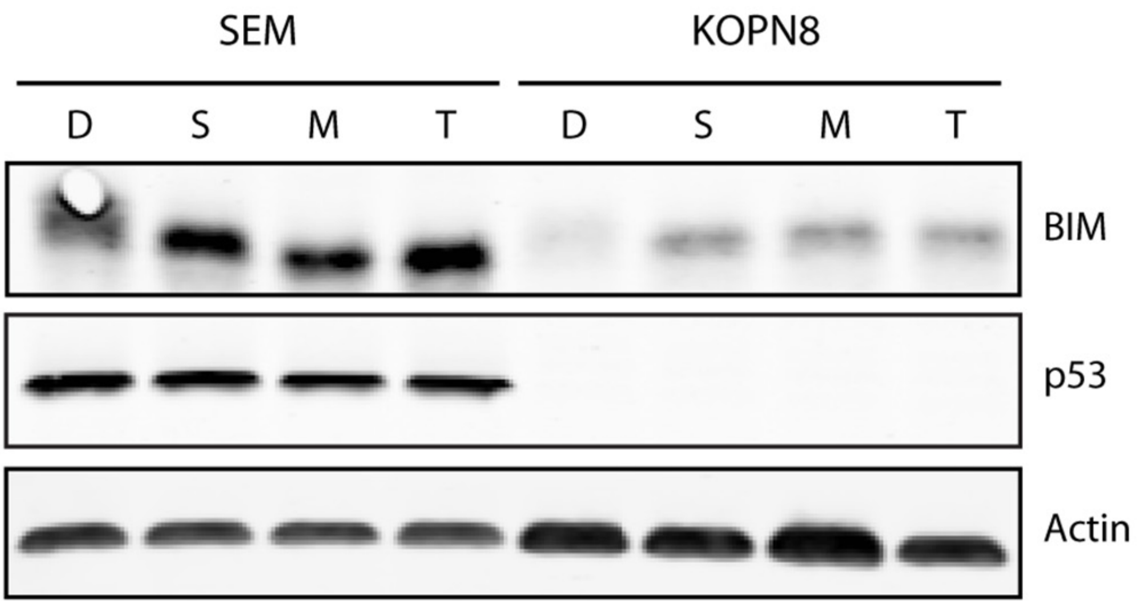

Supplementary Figure 5: BIM and p53 immunoblots of MEKi treated cells. Western blots of SEM and KOPN8 exposed for 48 hours to DMSO (D), Selumetinib (S), MEK162 (M) or Trametinib (T), for determination of BIM and p53 protein levels. Actin was used as loading control.

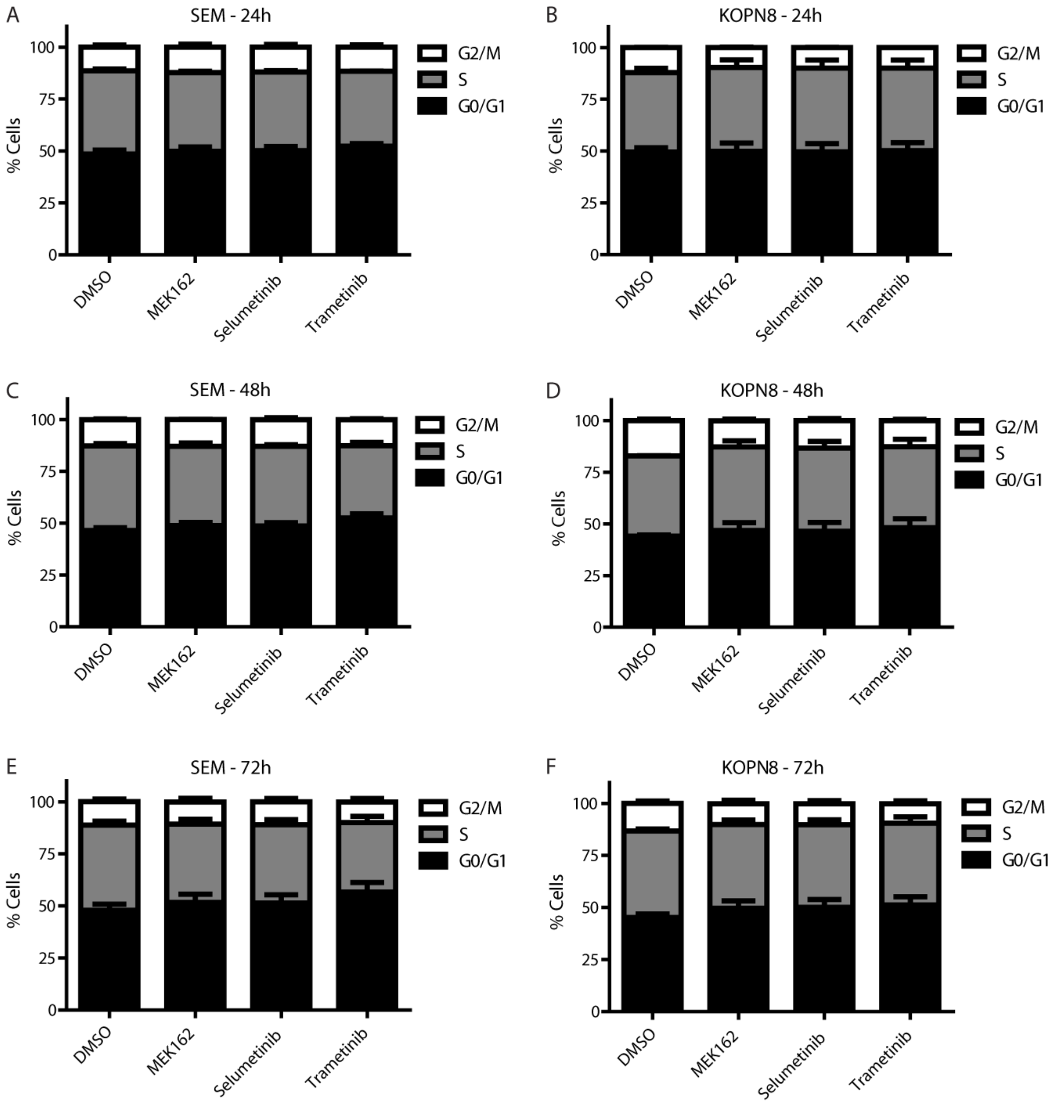

Supplementary Figure 6: Cell cycle progression after MEK inhibitor exposure. Representative graphs of percentages SEM **A, C** and **E.** or KOPN8 **B, D** and **F.** cells in G2/M (white), S (grey) or G0/G1 (black) cell cycle stages, as analyzed by DNA staining. Cells were exposed to vehicle control (DMSO) or 500 nM MEK162, Selumetinib or Trametinib for 24, 48 or 72 hours (A and B, C and D, E and F, respectively).

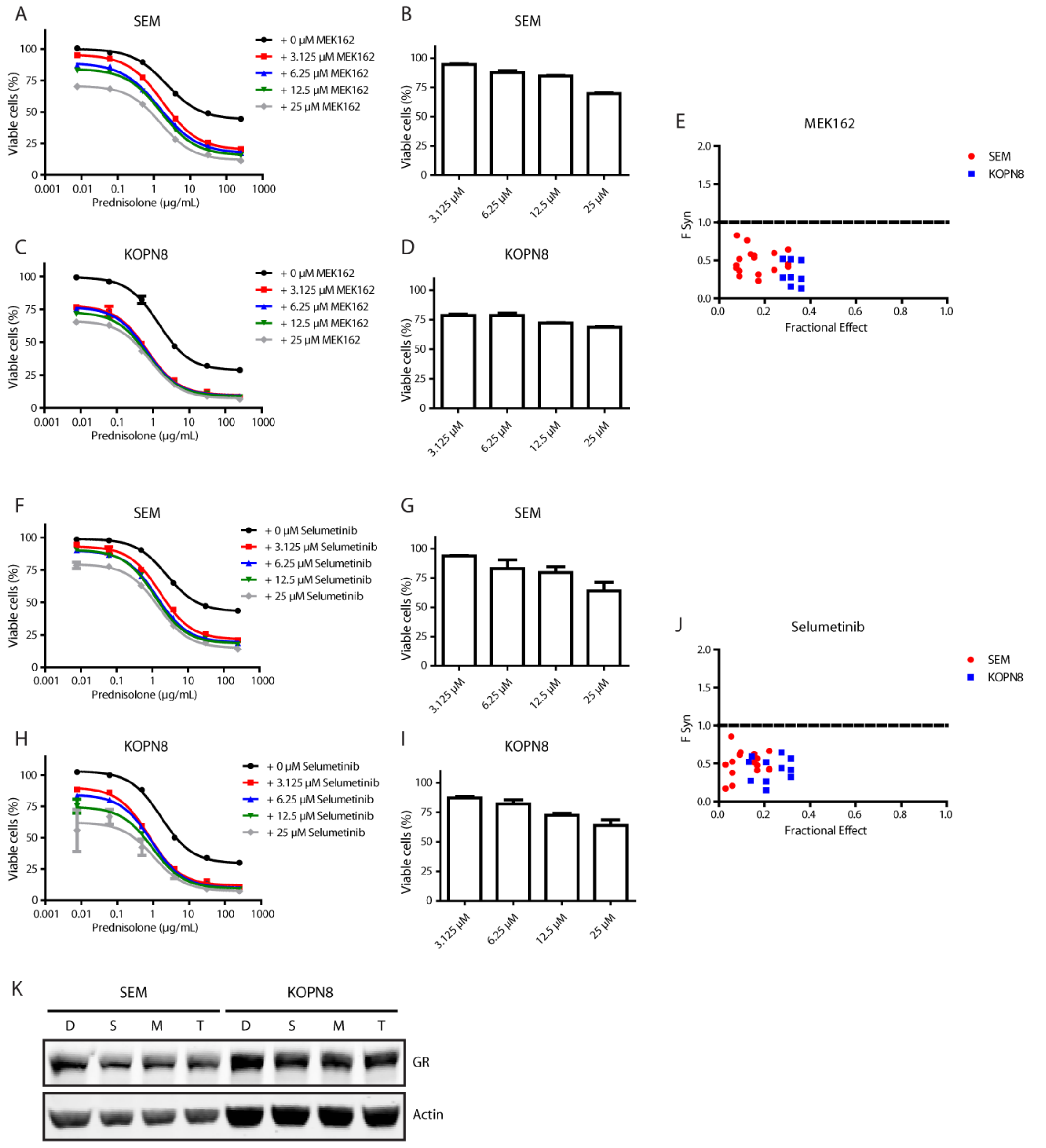

Supplementary Figure 7: Drug combinations of MEK162 and Selumetinib with prednisolone. A. Dose-response curves of the SEM cell line exposed to prednisolone alone (black curve) or in combination with 3.125 µM, 6.25 µM, 12.5 µM or 25 µM MEK162 (red, blue, green and grey curves, respectively). Low concentrations of MEK162 sensitize cells towards prednisolone. **B.** Response of SEM to the single MEK162 concentrations used in A. **C.** Dose-response curves of KOPN8 treated with prednisolone (black curve), or in combination with the aforementioned MEK162 concentrations (shown in red, blue, green and grey, respectively). KOPN8 cells are also sensitized towards prednisolone by co-exposure with low concentrations of MEK162. **D.** KOPN8 exposed to single MEK162 concentrations. **E.** Combined exposure to prednisolone and MEK162 (n=3) was quantified using F_{Syn} calculations ($F_{Syn}<1$ indicates synergy) and plotted against fractional effect (i.e. inhibition of cell viability). In both SEM (red) and KOPN8 (blue), moderate to strong synergy was observed. **F.** Dose-response curves of the SEM cell line exposed to either prednisolone alone (black), or in combination with Selumetinib (at the same concentrations used for MEK162). Low concentrations of Selumetinib can sensitize SEM cell towards prednisolone. **G.** Effect of different single Selumetinib concentrations in SEM. **H.** Combined exposure to prednisolone and low Selumetinib concentrations also sensitizes KOPN8 cells. **I.** Response of KOPN8 towards single concentrations of Selumetinib. **J.** Combinatorial effect of prednisolone and Selumetinib co-exposure (n=3) was quantified using F_{Syn} calculations. Moderate to strong synergistic effects between prednisolone and Selumetinib are observed for both SEM (red) and KOPN8 (blue). **K.** Western blot of SEM and KOPN8 exposed for 48 hours to DMSO (D), Selumetinib (S), MEK162 (M) or Trametinib (T), detecting glucocorticoid receptor (GR) and loading control (Actin).
